# Attention deficit hyperactivity disorder (ADHD) and premature mortality: A narrative review of the literature

**DOI:** 10.1007/s00787-026-03156-7

**Published:** 2026-08-06

**Authors:** Yasmin Rahali, Charlotte A. Dennison, Anita Thapar, Ajay K. Thapar

**Affiliations:** 1 Wolfson Centre for Young People’s Mental Health, Cardiff University, Hadyn Ellis Building, Maindy Road, CF24 4HQ Cardiff, UK; 2 Centre for Neuropsychiatric Genetics and Genomics, Division of Psychological Medicine and Clinical Neurosciences, School of Medicine, Cardiff University, Cardiff , UK

**Keywords:** ADHD, Premature mortality, Neurodevelopmental conditions, Stimulant medication, Psychiatric comorbidity

## Abstract

Attention-deficit hyperactivity disorder (ADHD) is associated with a higher risk of premature mortality compared to the general population, although reported risks vary. The aim of this paper was to review studies that have examined associations between premature mortality and ADHD and consider factors that could explain variation in risk estimates. A literature search was carried out using online databases (PMC, OVID, and Scopus library) to identify relevant articles. We then considered: sample ascertainment, sample size, method of diagnosing ADHD and age at diagnosis, sex, age range for mortality data, hazard ratio/ mortality rate ratio (95% confidence interval), sample size, and cause of death, as factors that could explain variation in risk estimates. We focus on nine papers that report risk of all-cause mortality in population samples via hazard ratios, odds ratios, and mortality ratios, which varied from 1.07 to 4.44. Higher risk estimates were found in studies that followed up to older ages and among individuals who were female, diagnosed with ADHD later, and had psychiatric comorbidity. Most follow up found non-natural causes (e.g. suicide, accidents) to be the most prevalent cause of death but studies with longer follow up suggested that non-natural causes may be more important in older individuals. Evidence suggests that ADHD medication reduces the risk of premature mortality but findings are clearer for stimulant than for non-stimulant medication. Clinicians need to be aware that females with ADHD, those with a delayed ADHD diagnosis and who show psychiatric comorbidity are at highest risk of premature mortality. However prescribing ADHD medication may reduce this risk.

## Background

ADHD is a neurodevelopmental condition that affects around 5% of children and adolescents [1, 2]. Although ADHD typically first presents in childhood, globally it is estimated that around 3.7% of adults live with the condition [3]. Many of the symptoms and difficulties associated with ADHD persist into adulthood and can impact day-to-day life-socially, physically, cognitively and psychologically [4] resulting in a variety of adverse functional outcomes.

Several large whole-population studies have highlighted that ADHD is associated with premature mortality [5–7]. A recent systematic review and meta-analysis identified a 2.18-times increased risk of mortality for people with ADHD [8]. The most recent study based on UK health records suggested that adults with ADHD have a shortened life expectancy of around seven to nine years [9]. This elevated risk of premature mortality is concerning. Core features of ADHD, such as impulsivity, may lead to higher rates of accidental death, but higher mortality may also be driven by lifestyle and cardiovascular risk factors including smoking, hypertension, and obesity [10].

However, reported risks vary considerably across different studies. In their systematic review, Catalá-López et al. highlight significant heterogeneity across studies and low confidence in the risk estimates [8]. This may be due to sample ascertainment and age of sample, because most studies have only been able to include follow up to early adult life. Some studies also have reported sex differences in rates of premature mortality and variation in rates of psychiatric comorbidity. Another source of variation is type of ADHD treatment provided, as a few recent studies have suggested that ADHD medication reduces premature mortality rates [11, 12].

We aimed to complement and expand upon the systematic review through a narrative synthesis of the literature, evaluating whether risk estimates for premature mortality varied by sample ascertainment, age at follow up, psychiatric comorbidity, and sex. We also assessed all-cause versus specific-cause mortality. Finally, we reviewed quasi-experimental studies to assess the impact of ADHD medication on premature mortality. We evaluate the literature included in the recent systematic review [8], as well as relevant studies that did not fall within its remit or have been published since.

## Methods

The literature was reviewed using an electronic database search of PubMed Central, OVID, and Scopus library. Search results were repeated using an AI tool (Elicit). Elicit is a literature search tool designed for scientific research, and we used it to identify any studies that may have been missed by our database searches. However, no studies were identified through Elicit that had not already been identified through our manual database searches. The search criteria for each database are outlined in supplementary materials.

The papers identified as being potentially relevant were then screened to determine whether (1) the target population comprised individuals diagnosed with ADHD or if there was a clear ADHD subgroup that also included comparison to the general population; (2) the exposure parameters, that is, ADHD diagnosis and method of diagnosis were specified (clinical diagnosis, from medical records, or self-report); (3) there was a robustly defined measure for the outcome of all-cause mortality; (4) the study design parameter focused on cohort studies or population registry-based studies and not case reports or narrative reviews.

After initial screening, the papers were reviewed in greater depth to judge if they were relevant to the aim of this review. We focus on those papers that (1) measure ADHD as the key exposure rather than a comorbidity, (2) report all-cause mortality as the outcome rather than a specific cause of mortality such as suicide, and (3) are based on population cohorts and population health record data so derive risk estimates applicable to the general population. The most recent search across all three databases was conducted in April 2025. Searches and manuscript screening were completed by YR, then separately repeated by AKT to ensure the final studies met the inclusion criteria and that no studies were incorrectly excluded. All authors agreed on which papers to include in this review. Our work was formulated and written against the Scale for the Assessment of Narrative Review Articles (SANRA) [13].

### Overview of included studies

**Fig. 1:**
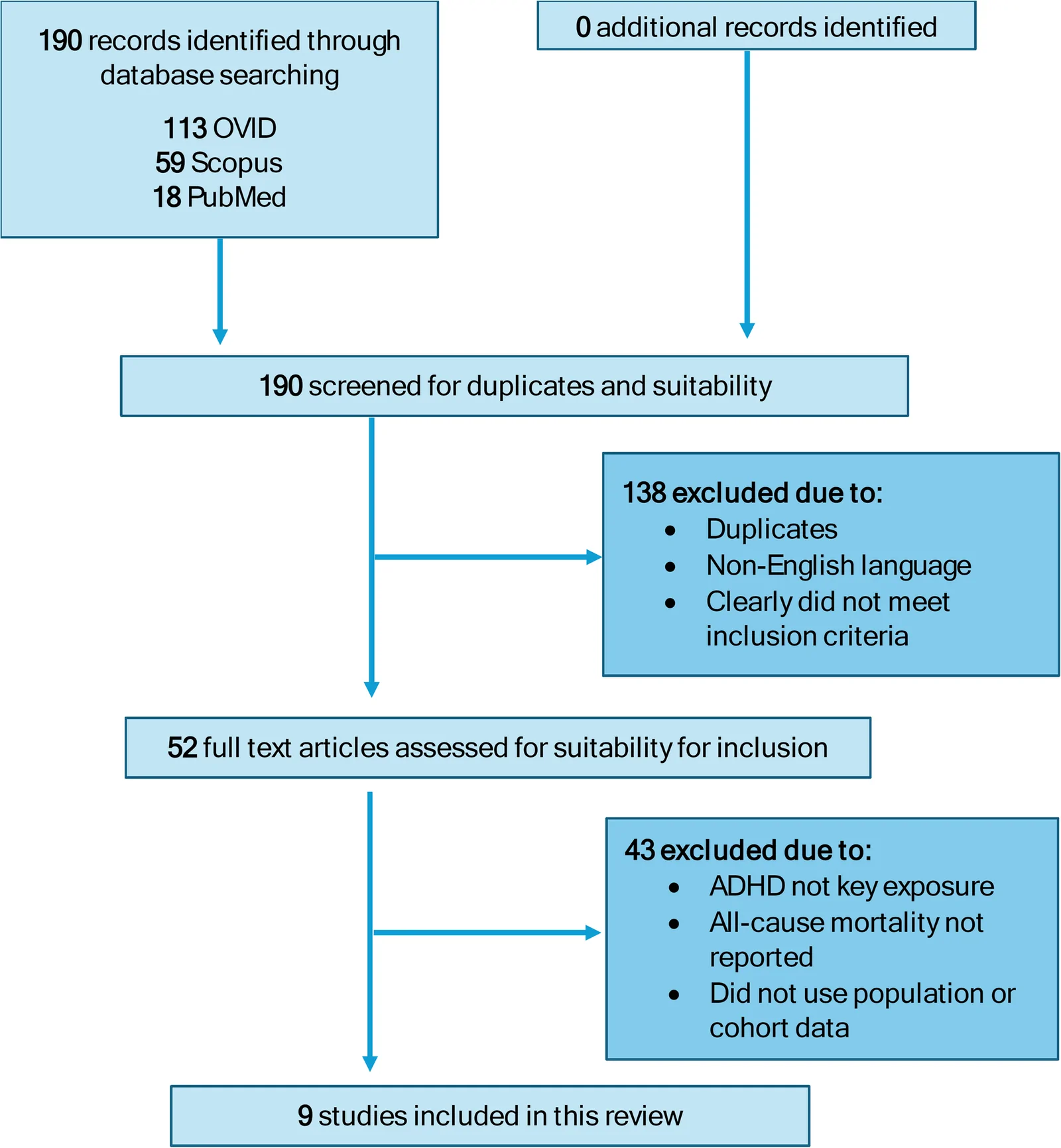
Flow diagram depicting the search strategy and inclusion and exclusion of studies for the review

Nine studies from Denmark, Sweden, Finland, UK, Taiwan, and USA fulfilled our inclusion criteria (Table 1). Figure 1 is a flow diagram summarising the study selection. Table 1 summarises the key features of included studies.

**Table 1:** Key features of included studies including risk estimates. dates are presented in DD.MM.YYYY format. HR=Hazards Ratio, MRR=Mortality Rate Ratio, OR=Odds Ratio, b=beta, SMR=Standardised Mortality Ratio

Paper	Location	Sample	Sample size	ADHD assessment	Age at follow up	Effect size (95% CI)	Model information
Barbaresi et al. [14]	USA	Birth cohort born 01.01.1976–31.12.1982	ADHD *n* = 367 Controls *n* = 4946	Recorded diagnosis of ADHD or evidence from various sources that child meets DSM-IV criteria.	Mean age 30y, range 27-34y	SMR = 1.88 (0.83–4.26)	Unadjusted, controls matched for age and gender
Dalsgaard et al. [6]	Denmark	Population cohort born 01.01.1981–31.12.2011	ADHD *n* = 32,061 Controls *n* = 1,890,187	Linked healthcare records. ICD-8 and ICD-10 diagnoses.	Maximum 32y	MRR = 2.44 (2.00-2.94)	Unadjusted
						MRR = 2.07 (1.70–2.50)	Adjusted for age, calendar year, sex, parental psychiatric history, parental age at birth, parental education, parental employment
London et al. [7]	USA	Adults completing 2007 National Health Interview Survey	ADHD n = ~ 654 Controls n = ~ 22,698	Self-reported ADHD diagnosis	18-85y	OR = 1.85 (1.06–3.23)	Adjusted for age
						OR = 1.78 (1.01–3.12)	Adjusted for age and sociodemographic variables
Chen et al. [5]	Taiwan	All Taiwanese residents aged 4–44 between 2000–2012	ADHD *n* = 275,980 Controls *n* = 1,931,860	ICD-9 record of ADHD between 01.01.2000 and 31.12.2012	4-44y	HR = 1.42 (1.31–1.54)	Unadjusted, controls matched for age and sex
						HR = 1.07 (1.00-1.17)	Adjusted for residence, income, outpatient visits past year before index date, congenital anomalies, and various neurodevelopmental and psychiatric comorbidities. Controls matched for age and sex.
Sun et al. [15]	Sweden	Population cohort born 01.01.1983–31.12.2009	ADHD *n* = 86,670 Controls *n* = 2,588,945	Linked healthcare records. ICD-10 diagnosis or stimulant prescription.	Maximum 31y	HR = 4.44 (3.95–4.98)	Adjusted for sex and year of birth
						HR = 3.94 (3.51–4.43)	Adjusted for sex, year of birth, birthweight, maternal age at birth, parental education, parental employment
London et al. [16]	USA	Adults completing 2007/2012 National Health Interview Survey	ADHD n = ~ 1919 Controls n = ~ 54,514	Self-reported ADHD diagnosis	18–80 + y	b = 0.75 (0.34–1.16)	Adjusted for age and sociodemographic variables
Schiavone et al. [17]	Finland	Population cohort born between 1971–1974 from a single maternity unit.	ADHD *n* = 115 Controls *n* = 508	Prospective assessment of symptoms via questionnaire. Diagnosis made against DSM-IV criteria.	Maximum 46y	HR = 2.53 (1.21–5.28)	Unadjusted
						HR = 2.15 (1.02–4.54)	Adjust for sex
Jeng et al. [18]	Taiwan	All Taiwanese residents from 2003–2017	ADHD *n* = 233,886 Controls *n* = 935,544	At least two ICD-9 or ICD-10 records of ADHD diagnosis	Maximum 67y	HR = 1.45 (1.30–1.61)	Adjusted for birth year, sex, income, urbanicity, and comorbidity
O’Nions et al. [9]	UK	Population cohort from primary care records	ADHD *n* = 30,039 Controls *n* = 300,390	Healthcare records, diagnosis of ADHD	18-94y	Males HR = 1.89 (1.62–2.19) Females HR = 2.13 (1.79–2.53)	Controls matched for age, sex, and primary care practice

## All-cause mortality

**Fig. 2:**
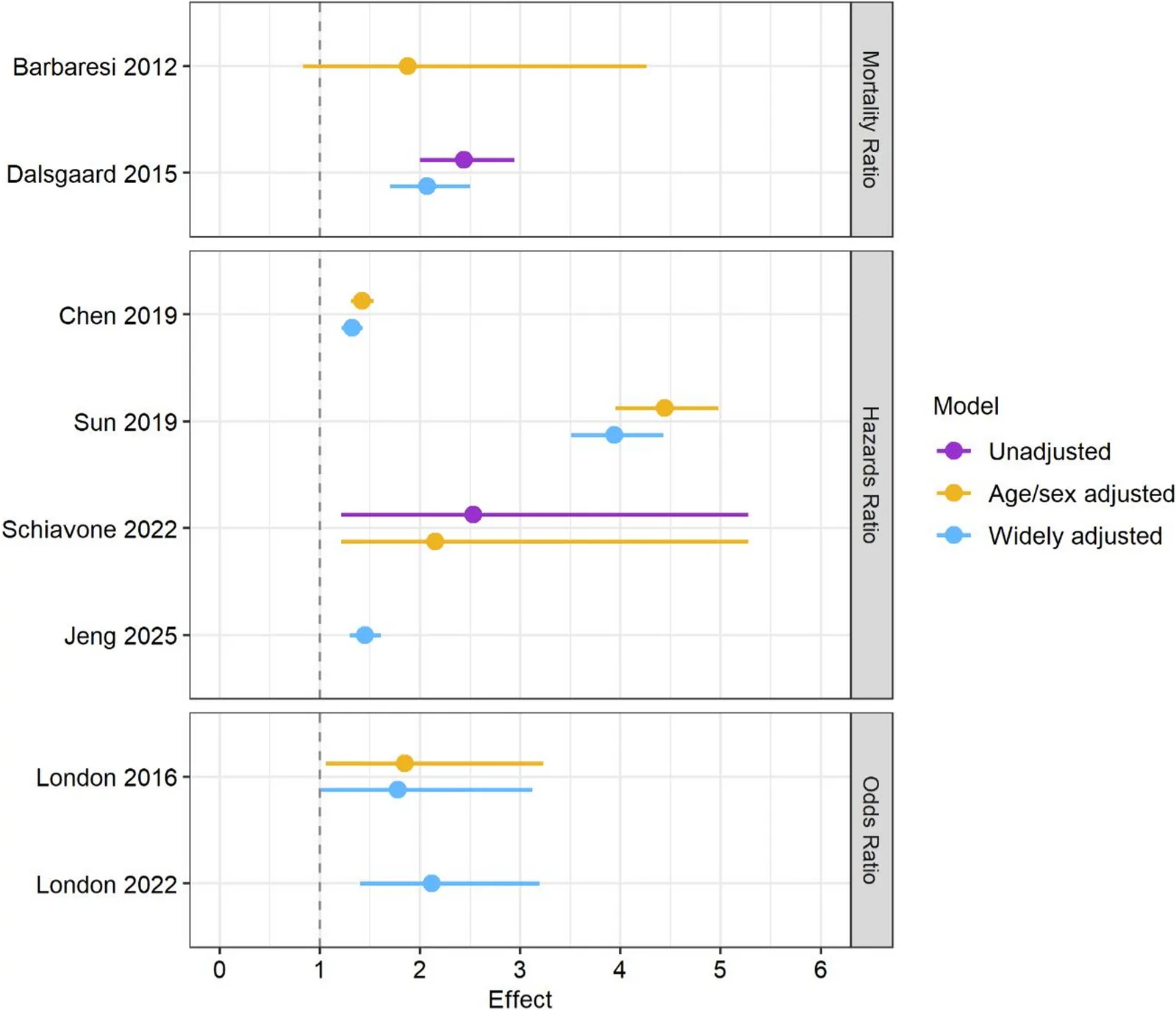
Association between ADHD and all-cause mortality across studies, grouped by type of effect size reported. Colour indicates covariates adjusted for within the model: ‘unadjusted’ refers to no covariates, ‘age/sex adjusted’ refers to only age and/or sex being accounted for, and ‘widely adjusted’ refers to models including age and/or sex plus additional covariates. For specific covariates included in each paper, see Table 1. NB: The findings of O’Nions et al. [9] are not presented in this figure as they are stratified by sex and are displayed in Fig. 3

All but one of the nine studies (see Table 1) found an increased risk for all-cause mortality amongst individuals with ADHD, summarised in Fig. 2. Reported risk estimates varied, as did the metric used to report risk. Hazards Ratios (HRs), Odds Ratios (OR), and Relative Risk (RR) all have similar interpretations in that an effect size of 1 equates to no differences between groups, whilst an effect greater than 1 indicates an increasing chance of the outcome in the exposed group. Although the terms OR and RR are often, and incorrectly, used interchangeably, they are not identical metrics, the former being a ratio of odds and the latter a ratio of probabilities in the ADHD versus the non-ADHD group. Some studies report a standardised mortality ratio (SMR), which is slightly different, as it is based on a reference population, and is comparable to a Mortality Ratio (MR). For an in depth discussion of the differences in the calculation and interpretation of each metric, we suggest George et al. [19]. Of those studies reporting a Hazards Ratio (HR: the most reported metric), estimates for all-cause mortality at any age ranged from 1.07 to 4.44. As expected, estimates were typically higher for unadjusted or minimally adjusted models (i.e. adjusted for age and/or sex), whilst lower estimates typically reflected greater adjustment for potential confounders.

The highest hazard ratio for mortality reported at any age was 4.44 (95% CI = 3.94–4.98), which reduced to 3.94 (95% CI 3.51–4.43) after accounting for a range of additional covariates, as reported by Sun et al. [15] using nationwide registry data in Sweden. The lowest hazard ratio for all-cause mortality at any age was 1.07 (95% CI = 1.00-1.17), reported by Chen et al. [5] after adjusting for numerous potential confounders including psychiatric comorbidity, using registry data from Taiwan.

## Age at follow-up and age at diagnosis

Findings suggested that age at follow up is an important factor in accounting for variability as the risk of mortality varied by age at follow up, with most, but not all, papers suggesting increasing risk with age. London and Landes [16] reported increased risk of mortality between ages 33 and 75 years, but not earlier or later in life. In an earlier paper however the same authors discussed how the majority (80.4%) of individuals with ADHD in their sample were aged 18–49 years [7]. 81.1% of the reported deaths, however, were concentrated amongst those aged 50 and older. Sun et al. [15] found a similar age-pattern risk, demonstrating a higher HR in adulthood (HR = 4.64 [95% CI 4.11–5.25]) compared to childhood (HR = 1.41 [0.97–2.04]). In contrast, Schiavone et al. [17] found risk of premature mortality to be elevated before age 30 (HR = 6.20 [1.78–21.57]), but not after age 30 in a Finnish study (HR = 2.70 [0.79–9.25]).

Age at first ADHD diagnosis also appears to be of relevance when considering increased mortality risk. A later ADHD diagnosis, after age 17, was associated with a 4–5 times increased risk compared to those without ADHD [6, 15]. In the Swedish study, all-cause mortality risk significantly increased the later a diagnosis was first received (adjusted HRs for ≤ 12 years = 1.33 [0.92–1.92]; 13–17 years = 2.41 [1.97–2.96]; ≥18 years = 9.12 [7.88–10.55]) [15]. This was consistent with findings from Denmark where diagnoses made later than age 17 were associated with a greater mortality rate ratio than those who were diagnosed in childhood and adolescence (adjusted MMRs for ages 1–5 = 1.86 [0.93–3.27]; ages 6–17 = 1.58 [1.21–2.03]; age ≥ 18 = 4.25 [3.03–5.78]) [6]. The authors commented that this could be due to the higher proportion of “persistent ADHD” in the sample causing more severe impairment [6]. This finding is important given that access to ADHD care is limited across most countries and there is evidence of an unmet need amongst those with ADHD [20]. Indeed, rates of ADHD identification and treatment may be higher in Scandinavian countries and several studies included in this review were based on Scandinavian registry data, so it is possible that mortality risks may be higher in other nations where healthcare provision for ADHD may be poorer. The identification and treatment gap needs to be better met in many countries and could help address this concerning reduction in life expectancy and increasing mortality risk. Timely identification of ADHD is a problem in many parts of the world, including the UK, especially for females and those from minority groups [21, 22]. Unfortunately, the studies we examined did not investigate minority groups separately. Delays in identification and its association with premature mortality is especially important given findings that suggest treatment maybe protective (discussed below). Although the waiting times for an ADHD diagnosis are at an all-time high in some countries, including the UK, early identification and intervention in schools and primary care may help address the inequalities in life expectancy for people with ADHD.

## Specific-cause mortality

Table 2 summarises associations between ADHD and mortality split by natural (medical conditions e.g. heart disease, stroke) and unnatural causes (e.g. suicide, accidents) of death, with unnatural causes further separated by specific cause where studied. Some studies highlighted that the risk of premature death of those with ADHD was significantly higher than the general population due to both natural and unnatural causes, whilst others did not find an association between ADHD and risk of death by natural causes [5, 17]. Although many reported that the risk of premature mortality was mainly attributed to non-natural causes such as accidents and suicide, most of these were cohort studies with mortality follow up only into early adulthood. When looking at the specific breakdown of unnatural causes, whilst unintentional injury was an overall more common cause of death, the relative risk of death by suicide was higher. ADHD was associated with a 1.78–8.63 increased hazard of death by suicide and a 1.30–3.94 increased hazard of death due to unintentional injury.

**Table 2:** Association between ADHD and specific causes of mortality. HR= Hazards Ratio, MMR= Mortality rate ratio. ‘Adjusted for’ refers to covariates included in each model

Paper Adjusted for	Natural causes (95% CI)	Unnatural causes (95% CI)	
Barbaresi 2013 Controls matched for age and sex	Unspecified	Suicide	SMR = 4.83 (1.14–20.46)
		Accidental Injury	SMR = 1.70 (0.49–5.97)
Dalsgaard 2015 Age, calendar year, sex, parental psychiatric history, parental age at birth, parental education, and parental employment	MMR = 1.70 (1.11–2.47)	Any unnatural cause	MMR = 2.40 (1.81–3.13)
Chen 2019 Sex, age, residence, income, outpatient visits 1 year before index date, neurodevelopmental and psychiatric comorbidity, and other cause mortality through competing risk model	HR = 0.91 (0.80–1.15)	Suicide	HR = 2.09 (1.62–2.71)
		Accidental Injury	HR = 1.30 (1.10–1.52)
Sun 2019 Year of birth, sex, birthweight, maternal age at birth, parental education, and parental employment status	HR = 2.47 (1.66–3.68)	Any unnatural cause	HR = 6.48 (5.12–8.21)
		Suicide	HR = 8.63 (6.27–11.88)
		Accidental Injury	HR = 3.94 (2.49–6.25)
Schiavone 2022 Sex	HR = 1.11 (0.23–5.32)	Any unnatural cause	HR = 2.82 (1.12–7.12)
Jeng 2025 Sex, birth year, income, urbanisation, and physical and psychiatric comorbidities	HR = 1.26 (1.06–1.49)	Any unnatural cause	HR = 1.82 (1.61–2.07)
		Suicide	HR = 1.78 (1.36–2.32)
		Accidental Injury	HR = 1.73 (1.48–2.02)

## Psychiatric comorbidity

Psychiatric comorbidity is common in ADHD, including substance use disorders, depression, anxiety, personality disorders, and bipolar disorder [23]. There is consistent evidence that psychiatric comorbidity increases the risk of all-cause mortality in people with ADHD [6, 15, 18]. Evidence also suggests a dose-response relationship between increasing numbers of psychiatric comorbidities and mortality risk in ADHD, with Sun et al. [15] reporting a HR for all-cause mortality of 25.22 for individuals with ADHD and four or more comorbidities, compared to 1.41 for ADHD-only. Whilst comorbidity across a range of psychiatric conditions has been associated with increased risk of mortality, substance use disorder (SUD) in particular has been associated with the highest risk of mortality in ADHD (HRs 3.66–8.01 for ADHD + SUD vs. ADHD-only) [15, 18]. Timing of psychiatric comorbidities may also be an important factor, as there is some evidence that early-onset comorbidities increase the risk mortality due to natural causes, whilst later-onset comorbidities were associated more strongly with unnatural causes of death [15]. Psychiatric comorbidity impacts on the choice of treatment in ADHD [24], and also increases rates of medication switching and discontinuation [25, 26], which could further impact the relationship between ADHD, psychiatric comorbidity, and mortality.

### Sex differences

**Fig. 3:**
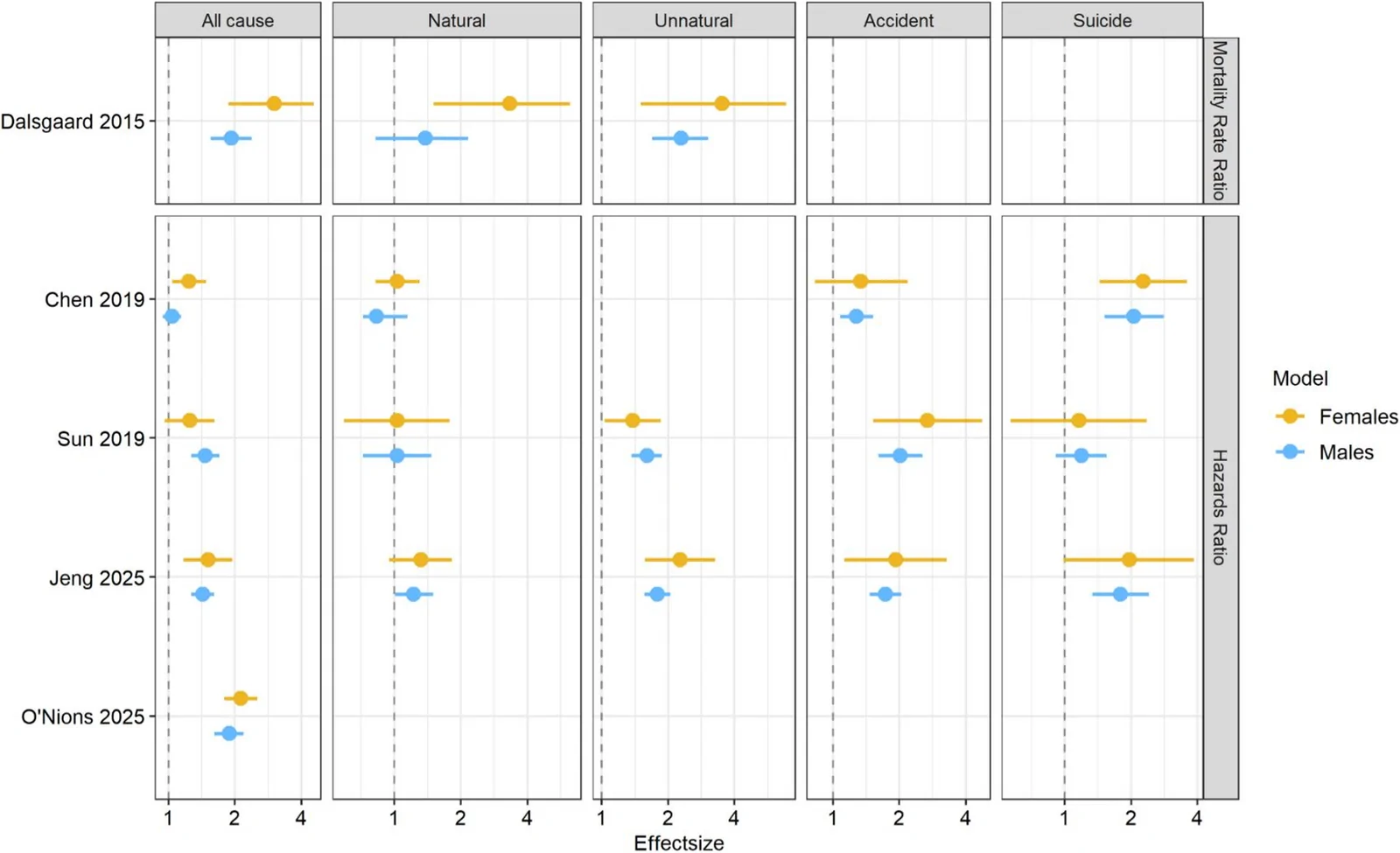
Sex differences in the association between ADHD and all cause and specific causes of mortality. Points refer to Hazards Ratio or Mortality Rate Ratio, and error bars refer to 96% confidence intervals. X axis is presented on a log10 scale. Orange indicates association in females, whilst blue indicates association in males. Estimates are given from the most completely adjusted model in each paper

Only some of the papers reviewed specifically reported differences in mortality risk by sex. These findings are shown in Fig. 3. Most papers found that females with ADHD had an increased risk of all-cause mortality compared to males with ADHD, although confidence intervals overlapped in all papers reporting sex differences. In the most recent UK national records-based study, a similar pattern of results was found with a life expectancy reduction of 6.78 years in males and 8.64 years in females with ADHD, compared to matched controls [9].

Differences in mortality rates may be explained by rates of psychiatric comorbidity varying between males and females with ADHD. In a Swedish health record study, the authors reported a higher risk of all-cause mortality in females with ADHD than males (females HR = 6.46 [5.13–8.14]; males HR = 5.00 [4.40–5.67]), that substantially reduced when adjusting for neurodevelopmental and psychiatric comorbidity (females HR = 1.25 [0.96–1.62]; males HR = 1.47 [1.27–1.70]) [15]. Similarly, studies adjusting for psychiatric comorbidity reported a smaller degree of difference in mortality rate between males and females compared to studies that did not adjust for psychiatric comorbidity [6, 18] (Fig. 3).

Studies of younger populations (up to age 30) generally found that all-cause mortality was higher in females and death by suicide was a particular risk. For older individuals the reasons for the increased risk in females are less studied but several studies suggest that this increased mortality risk may be partly related to higher rates of co-morbid psychiatric conditions such as depression [27]. Other studies however have not observed the same pattern of findings relating to sex differences. One study of adults with persistent ADHD found marginal differences in prevalence of psychiatric comorbidity between males and females [28].

Another potential reason for this sex disparity may arise from the potential delay in diagnoses of ADHD in women. On average, females are diagnosed and treated four years later than their male counterparts [29] and we observed that a later diagnosis of ADHD was associated with higher mortality risk. Referral bias for women may play a role as the diagnostic criteria for ADHD is historically “male symptom” focussed. Female patients with ADHD have been found to be referred for further evaluation less often than male patients, which may be due to under recognition or diagnostic overshadowing [30]. Therefore, women who are referred and receive a diagnosis of ADHD may present with more severe and impairing symptoms and problems, thus accounting for greater premature mortality risk. In addition, women with ADHD are less likely to receive medication than males, unless they have “prominent, externalising issues”, which may also contribute to their higher mortality risk [31, 32].

## Effects of ADHD medication on mortality rates

Rates of ADHD identification and treatment vary widely across countries and between different regions within the same country. As an addition to our core literature review, our search further identified an additional three quasi-experimental studies that examined the effects of ADHD medication on all-cause mortality [11, 12, 33]. These studies were deemed relevant to this paper as ADHD medication is another factor that could contribute to varying estimates of premature mortality risk. ADHD medication use was associated with reduced all-cause mortality in all three studies (HR range 0.61–0.89). Type of medication may be an important factor, as Vasiliadis et al. [11] found a reduced risk of mortality with stimulant prescription but not with non-stimulant prescription. However, non-stimulant prescription was comparatively rare and is not first-line treatment [34]. The effects of non-stimulant only and combined stimulant and non-stimulant pharmacotherapy on mortality rates requires further investigation.

Some papers reported on specific causes of death [12, 33], with neither of these studies observing an association between medication use and death due to natural causes. Li et al. [12] reported that initiating methylphenidate was associated with reduced risk of unnatural causes of death, such as suicide and accidental injury. They hypothesised that this was due to the alleviation of some of the core ADHD symptoms such as impulsivity, but also, that there was a potential knock-on effect of reducing the risk of developing psychiatric co-morbidities. After controlling for confounders, Chen et al. [33] did not identify a reduction in specific-cause mortality with methylphenidate use, although they note limited power to identify specific cause-of-death effects. One study, focussed specifically on suicide, did not observe an association between stimulant or non-stimulant medication and completed suicide [35], although another did observe a reduction in suicide attempts with stimulant medication [36].

The results of the studies on medication and all-cause mortality are summarised in Table 3, and highlight that treatment is another likely contributor to variation in reported risks of premature mortality in people with ADHD. Although caution is needed in drawing firm conclusions as these studies were quasi-experimental rather than randomised controlled trials, they suggest that risks of premature mortality could be reduced with timely recognition and treatment.

**Table 3:** Association between medication use and all-cause mortality in people with ADHD

Paper	Location	Sample size	Medication studied	Follow-up period	Adjusted hazards ratio (95% CI)
Chen et al. [33]	Taiwan	136,192	Methylphenidate	3–13 years	All cause HR = 0.81 (0.67–0.98)
					Unnatural cause: HR = 0.72 (0.49–1.05)
					Natural cause: HR = 0.85 (0.68–1.05)
Li et al. [12]	Sweden	148,578	Methylphenidate, amphetamine, dexamphetamine, lisdexamfetamine, atomoxetine, and guanfacine	2 years	All cause: HR = 0.79 (0.70–0.88)
					Unnatural cause: HR = 0.75 (0.66–0.86)
					Natural cause: HR = 0.86 (0.71–1.05)
Vasiliadis et al. [11]	Canada	217,192	Amphetamine and methylphenidate-based stimulants, atomoxetine, and guanfacine	Up to age 25	All cause HR = 0.61 (0.48–0.76)

## Discussion

We aimed to synthesise the literature on ADHD and all-cause mortality to identify and evaluate factors that might account for variability in estimates of risk. We did not aim to conduct a meta-analysis as this has already been published recently [8]. We found consistent reports, across different countries, that ADHD is associated with higher premature mortality risk, but reported rates of risk were variable. Most found increased premature mortality risk from non-natural mechanisms such as accidental injury and suicide. However, findings varied by age at follow up.

We identified several factors that may contribute to this variability in risk estimates. Later age at diagnosis of ADHD was especially associated with higher risks [6]. Females with ADHD also seemed at higher risk of premature mortality than males, although this may in part be due to higher rates of psychiatric comorbidity [15]. Some study findings highlighted increased risks from comorbid psychiatric conditions and substance use regardless of sex [6, 18]. This may be especially important given that while ADHD is associated with premature mortality and a shortened lifespan (7–9 years in the UK) [9], severe mental illness, mood disorders, and personality disorders are themselves associated with 13–15 years reduction in life expectancy (based on a recent meta-analysis of worldwide studies) [37]. Quasi-experimental studies have highlighted that medication for ADHD, mainly stimulant medication, seems to reduce the risk of premature mortality so ADHD treatment may be another contributor to the variation in risk.

### Implications for clinical practice and service design

Two key findings that emerged were that later diagnosis of ADHD increased the risk of mortality, and that ADHD medication appears to reduce the risk of mortality. These findings strongly support the need for earlier recognition and treatment of ADHD as methods to prevent avoidable deaths. Later diagnosed and unrecognised ADHD are associated with widespread negative outcomes in addition to premature mortality, including higher rates of psychiatric conditions, substance misuse, suicide attempts, lower quality of life, criminality, and greater health service use [21, 38]. Furthermore, ADHD medication has been shown to reduce the rate of accidental injury [39], emergency department use [39], depression [40], substance misuse [41], unemployment [42], and criminality [43], as well as improve academic outcomes [44]. Combined, these studies underscore the urgent need for early recognition of ADHD to provide individuals with support and, where appropriate, treatment to prevent adverse outcomes. They further lend support to the use of prescription stimulants as a treatment that reduces ADHD-associated risks. Future research is needed to evaluate whether non stimulant medications and non-pharmacological intervention similarly reduces risks. Regardless, given the widespread economic, personal and family impacts of these outcomes, it seems imperative to invest in ADHD services, prioritising early and equitable access.

There is evidence to suggest that ADHD is associated with various physical co-morbidities, most commonly metabolic, vascular and respiratory [45] all of which could contribute to premature mortality from natural causes. However, it is difficult to make the distinction between whether these conditions occur as a direct result of ADHD or as a result of lifestyle factors at an individual level. Evidence suggests a combination of both, as ADHD impacts on potential health-risks such as cigarette smoking, obesity and substance misuse, contributing to a greater chance of developing certain conditions that carry a risk of early mortality [46, 47]. Recent work suggests that cigarette smoking and obesity mediate the link between ADHD and premature mortality [48]; both of these risk factors are modifiable [49]. Regardless, our findings highlight the need for more frequent, integrated healthcare assessments of physical health in those with ADHD to implement prevention and treatment strategies, helping to address inequalities in life expectancy.

### Strengths and limitations

All the papers reviewed had similar methodologies, were whole- population cohort studies or used national registry data which allowed for a large, diverse sample population. Importantly similar findings were observed across different countries. Schiavone et al.’s [17] sample however comprised only 839 individuals, limiting the power of the study. The London and Landes [7, 16] studies relied on self-reported answers regarding ADHD diagnoses, which may have impacted the reliability of their collated data due to under/over diagnosis/reporting in different subpopulations. Sun et al. [15] discussed their limitations surrounding ADHD diagnosis as they were defined from routine health care data rather than structured clinical interviews, potentially missing undiagnosed cases. Similarly, Schiavone et al. [17] relied on retrospective diagnoses, which may have under/overestimated the true prevalence of ADHD. Some studies concluded their follow up at a young age (participants aged around 30 years). This means it was not possible for such studies to consider the effect of ADHD in later life and leaving long enough for adequate representation and effective data collection. Deaths were reliably assessed across all studies using national registers/indexes.

It is worth noting that the ratio of males to females with ADHD across all studies reviewed was approximately 3:1. This may suggest an underrepresentation of the number of females with ADHD but also limits the ability to effectively conduct statistical analysis on the female population in each study due to a smaller sample size. However, one study [15] had almost equal numbers of males and females and a large sample. Although we examined three studies reporting on the effect of ADHD medication on premature mortality rates, none of the other nine studies in this review analysed the association between ADHD treatment and mortality, nor did they disclose whether participants were on medication. However, one study cohort was from an era prior to widespread ADHD medication use. A large proportion of participants across all papers may have been on ADHD medication as the majority of ADHD diagnoses were identified by either health records or evidence of ADHD medication being prescribed. Even with this assumption, it was impossible to determine whether participants were actively taking their medication at any given and hence whether medication would have affected the reliability of the conclusions drawn. Also, rates of ADHD medication prescribing vary widely between countries and within different regions of the same country which further increases uncertainty about the contribution of medication. Finally, the aim of this narrative review was not to conduct a meta-analysis as this has already been published recently [8]; rather it was to synthesise the literature to identify factors that might account for variability in estimates of risk.

### Future directions

Whilst there is good evidence linking ADHD to premature mortality, there are several questions remaining, particularly surrounding the use of pharmacotherapy and non pharmacological interventions including measures to reduce risks such as cigarette smoking and obesity to prevent untimely death. It is unclear whether there are key developmental stages during which treatment may be most effective, the extent to which treatment adherence and duration of use impacts on mortality, or if there are key groups for whom treatment may be effective. It would be especially valuable to have a greater understanding of which group of people with ADHD have a higher mortality risk, and the mechanisms through which sex differences in mortality risk arise. We speculate that a late diagnosis and psychiatric comorbidity may be key mechanisms explaining sex differences in ADHD-associated premature mortality, but further research is needed to fully understand how the developmental timing, presentation, and treatment of comorbid psychiatric conditions affects the risk of mortality in ADHD.

## Conclusions

Overall, variability in risk estimates of ADHD on mortality were fairly wide, and driven in part by heterogeneity in the age of diagnosis, length of follow-up, specific cause of mortality, sex, and rates of psychiatric comorbidity. Recognition and treatment of ADHD is not consistent around the world, and this will likely result in continued variation across the literature. Nevertheless, there was a clear and marked association between ADHD and premature mortality, and evidence to suggest that these risks are modifiable through treatment with stimulant medication.

## Data Availability

No datasets were generated or analysed during the current study.

## Declarations

### AI

During the preparation of this work the authors used Elicit in order to identify any studies that may have been missed by initial database searches, as described in the methods. After using this tool/service, the authors reviewed and edited the content as needed and take full responsibility for the content of the published article
